# Increased genomic burden of germline copy number variants is associated with early onset breast cancer: Australian breast cancer family registry

**DOI:** 10.1186/s13058-017-0825-6

**Published:** 2017-03-16

**Authors:** Logan C. Walker, John F. Pearson, George A. R. Wiggins, Graham G. Giles, John L. Hopper, Melissa C. Southey

**Affiliations:** 1 Mackenzie Cancer Research Group, Department of Pathology, University of Otago, Christchurch, New Zealand; 2 Biostatistics and Computational Biology Unit, University of Otago, Christchurch, New Zealand; 3 Cancer Epidemiology Centre, The Cancer Council Victoria, Melbourne, Australia; 4 Centre for Epidemiology and Biostatistics, Melbourne School of Population and Global Health, University of Melbourne, Melbourne, Victoria Australia; 5 Genetic Epidemiology Laboratory, Department of Pathology, University of Melbourne, Melbourne, Victoria Australia

**Keywords:** Breast cancer, Early onset, Copy number variants, Inherited susceptibility, Genome-wide association analysis

## Abstract

**Background:**

Women with breast cancer who have multiple affected relatives are more likely to have inherited genetic risk factors for the disease. All the currently known genetic risk factors for breast cancer account for less than half of the average familial risk. Furthermore, the genetic factor(s) underlying an increased cancer risk for many women from multiple-case families remain unknown. Rare genomic duplications and deletions, known as copy number variants (CNVs), cover more than 10% of a human genome, are often not assessed in studies of genetic predisposition, and could account for some of the so-called “missing heritability”.

**Methods:**

We carried out a hypothesis-generating case-control study of breast cancer diagnosed before age 40 years (200 cases, 293 controls) using population-based cases from the Australian Breast Cancer Family Study. Genome-wide scanning for CNVs was performed using the Human610-Quad BeadChip and fine-mapping was conducted using PennCNV.

**Results:**

We identified deletions overlapping two known cancer susceptibility genes, (*BRCA1* and *BLM)*, and a duplication overlapping *SMARCB1*, associated with risk. The number of deletions across the genome was 1.5-fold higher for cases than controls (*P* = 10^-16^), and 2-fold higher when only rare deletions overlapping genes (frequency <1%) were assessed (*P* = 5 × 10^-4^). Association tests of CNVs, followed by experimental validation of CNV calls, found deletions overlapping the *OR4C11* and *OR4P4* genes were associated with breast cancer (*P* = 0.02 and *P* = 0.03, respectively).

**Conclusion:**

These results suggest rare CNVs might have a role in breast cancer susceptibility, at least for disease at a young age.

**Electronic supplementary material:**

The online version of this article (doi:10.1186/s13058-017-0825-6) contains supplementary material, which is available to authorized users.

## Background

Breast cancer is the most common malignancy among women in the developed world, and is increasing rapidly in the developing world. A proportion of women with breast cancer have multiple affected relatives and are therefore more likely to have inherited genetic factors that increase their risk of developing the disease. All the currently known genetic risk factors for breast cancer currently only account for around 48% of average familial risk, and account for a lower proportion of the familial risk of disease at a young age, and the vast majority of women from multiple-case families do not have a known genetic explanation for their increased cancer risk [1, 2]. Thus, for a substantial fraction of women, including young affected women and those with a family history, the cause of their disease remains unexplained.

Copy number variants (CNVs) are estimated to cover 5–10% of the human genome [3] and, based on nucleotide coverage, are responsible for the majority of genetic variability in human populations. CNVs have been reported to disrupt genes known to be involved in breast cancer susceptibility, including *BRCA1*, *BRCA2*, *TP53* and *CHEK2* [4], and could similarly alter other genes involved in pathways related to breast cancer susceptibility. Furthermore, several array-based studies have reported candidate rare CNVs that overlap genes; variants in these might contribute to breast cancer susceptibility [5–7]. However, there has been a notable lack of consistency across these studies, probably because many women carry rare CNVs or because of false CNV calls caused by technical issues [8]. Evidence from some studies has suggested that the frequency and size of germline CNVs are increased in women with breast cancer [5, 7, 9], and that this might be strongest for CNVs that overlap gene regions [5, 7]. Thus, functional disruption of genes by CNVs across the genome might contribute to the genetic basis of breast cancer risk.

Only a few studies have examined the relationship between common germ-line CNVs and breast cancer risk. A large genome-wide association study (2000 breast cancer cases and 3000 controls) from the Wellcome Trust Case Control Consortium suggested that such CNVs were unlikely to have a major role in the genetic basis of breast cancer [10]. However, more recent genome-wide association studies of common CNVs (mean allele frequency (MAF) ≥5%) in Chinese and European women identified a deletion in the *APOBEC3* gene cluster associated with up to 1.3-fold and up to 2.3-fold increase in risk of breast cancer associated with hemizygous and homozygous deletions, respectively [11, 12].

To better understand the role of CNVs in breast cancer risk we have conducted a hypothesis-generating study of breast cancer at a young age (diagnosed before the age of 40 years). Our study aimed to assess whether CNVs across the genome are more frequent in such breast cancer cases when compared with unaffected controls, and whether cancer susceptibility genes are disrupted by rare CNVs.

## Methods

### Subjects

We conducted a hypothesis-generating study of 258 women who were diagnosed with breast cancer before the age of 40 years from the population-based Australian Breast Cancer Family Study [13–15]. These cases had been previously screened for germline mutations in *BRCA1, ATM, CHEK2, PALB2, TP53*, *BRCA2*, *CDH1*, and *FANCM* [13, 14, 16–25]. A total of 348 women unaffected with breast cancer (controls) were selected from participants in the Australian Mammographic Density Twins and Sisters Study, a cross-sectional study of twins and their sisters [15, 26]. All study participants provided written informed consent.

### Genotyping and identification of CNVs

All DNA samples were genotyped with the Human610-Quad BeadChip (Illumina, Inc, San Diego, CA, USA) with approximately 610,000 markers (including approximately 20,000 non-polymorphic markers) for single nucleotide polymorphism (SNP) and CNV analysis. Samples were processed using Illumina’s recommended protocol for Infinium HD assays. Data for each array were normalised using GenomeStudio 2011.1 software (Illumina). Probe information, including genomic location, signal intensity (Norm R), allele frequency (Norm theta), log R ratios (LRRs), and B allele frequencies (BAF), for each sample was calculated and exported from GenomeStudio. All samples had a call rate >95%. The CNV calls were generated using the PennCNV program (version 27 Aug. 2009), using the default program parameters, library files, and genomic wave adjustment.

Quality control procedures were performed to remove poor-quality array data (Additional file 1: Figure S1). Samples were excluded if they met the following criteria: log R ratio standard deviation >0.28; B allele frequency drift >0.01; waviness factor deviating from 0 by >0.04, or with the number of CNV calls exceeding 70. To reduce false positives, CNV calls were excluded if they contained <5 probes, and/or were ≥1000 kb in size. A total of 200 cases and 293 controls passed quality control steps and were assessed in the study. CNV data used in the study are shown in Additional file 2: Table S1.

### Identification of genes overlapping CNVs and defining rare CNVs

To avoid examining multiple isoforms from genes, we annotated 39,544 UCSC RefSeq (NCBI36/Hg18) transcripts using the SOURCE database [27] and defined the genomic intervals for a total of 18,791 unique genes. Thus, each gene interval encompassed the start and end of all associated RefSeq transcripts (Additional file 1: Figure S2). CNVs and gene regions that were estimated to overlap by at least one base pair were identified in a genome-wide scan using Intersect and Join tools from the Galaxy web server [28–30].

Because putative CNV calls do not typically conform to discrete genomic regions in different women, we used the genome coordinates of 18,791 RefSeq gene (NCBI36/Hg18) boundaries to define a CNV region (Additional file 1: Figure S1). Each of these regions therefore represented a cluster of one or more CNVs overlapping a well-characterised gene in the human genome and was used to measure the frequency of CNVs in our study. Rare CNVs were defined as those with a frequency <1% in the total sample.

### CNV validation by quantitative PCR

DNA samples were used to experimentally validate putative CNVs at 12 genomic regions using Human TaqMan® Copy Number Reference Assays (Thermofisher Scientific Inc). Primer and probe sequences are presented in Additional file 3: Table S2. RNaseP was used as an endogenous reference gene. All assays were carried out in triplicate.

### Statistical analysis of CNV load

Welch’s *t* test was conducted to establish the level of significance associated with the difference in CNV carrier frequencies between the cases and controls. This test is an adaptation of Student’s *t* test designed to cope with datasets that have unequal variances. The statistical package R version R 2.14.2 was used to perform statistical analyses. *P* values <0.05 were considered significant.

### Genome-wide CNV association analysis

Genes overlapping CNVs identified in cases or controls were assigned as having DNA loss (copy number states zero or one) or DNA gain (copy number states three or four). Perl 5.14.2 (ActiveState, Canada) was used to produce counts of the CNVs based on copy number state and the gene region by which they were defined. The CNV state with the most CNVs for each region was identified. A corresponding table of incidence of the most numerous CNV state in that region was analysed between cases and controls using Fisher’s exact test. This was used to calculate odds ratios, 95% confidence intervals and *P* values for the association between each CNV and cancer status. *P* values were adjusted for multiple testing with a false discover rate of 5%, using the method of Benjamini and Yekutieli (2001). Tests with a corrected *P* value <0.05 were considered statistically significant. The analysis was performed based on CNVs defined by their location within a gene region.

## Results

### CNV discovery in cases of early-onset breast cancer and in controls

A total of 58 cases and 55 controls were removed from the study after quality control criteria were applied, leaving 200 cases and 293 controls for downstream analyses (Additional file 1: Figure S1). Using PennCNV software, a total of 5109 and 6133 CNV calls were generated for cases and controls, respectively, ranging from 0.6 to 998 kb. The average number of CNVs observed in the two study groups was larger in cases than controls (25.6 vs. 20.9; *P* = 2 × 10^-10^) (Table 1). When accounting for copy number type, the average number of deletions was 1.5-fold greater in cases compared with controls (18.1 vs. 12.4; *P* = 2 × 10^-16^), whereas the average number of copy number gains in cases was slightly lower than that of controls (7.4 vs. 8.5; *P* = 0.01). These results suggest that women with early-onset breast cancer carried a greater CNV load across the genome compared with controls and that this feature was due to the inheritance or *de novo* formation of genomic deletions.

**Table 1:** Frequency of CNVs and overlapping genes in breast cancer cases and controls

Genomic feature	Total count		Mean frequency		Difference in means		Case/control ratio	*P* value
	Cases (*n* = 200)	Controls (*n* = 293)	Cases	Controls	Case - controls	95% CI		
CNVs								
All	5109	6133	25.6	20.9	4.6	3.2, 6.0	1.2	2e-10
Deletions	3622	3644	18.1	12.4	5.7	4.4, 7.0	1.5	2e-16
Gains	1487	2489	7.4	8.5	-1.1	-1.9, -0.3	0.9	0.01
CNVs overlapping genes								
All	1734	2069	8.7	7.1	1.6	0.9, 2.4	1.2	3e-05
Deletions	1048	956	5.2	3.3	2.0	1.4, 2.6	1.6	4e-10
Gains	686	1113	3.4	3.8	-0.4	-0.8, 0.1	0.9	0.1
Genes overlapping CNVs								
All	2816	3501	14.1	12.0	2.1	0.5, 3.8	1.2	0.01
Deletions	1450	1368	7.3	4.7	2.6	1.7, 3.5	1.6	2e-08
Gains	1366	2133	6.8	7.3	-0.5	-1.8, 0.9	0.9	0.5

*CNV* copy number variation

To assess the potential disruption of CNVs to functional regions across the genome, CNVs overlapping 18,791 reference sequence (RefSeq) genes (NCBI36/Hg18) were identified in cases and controls. Thirty-four percent of CNVs were predicted to overlap genes in both study groups (Table 1). Deletions affecting gene regions were shown to be 1.6-fold more frequent in cases compared to controls (5.2 vs. 3.3; *P* = 4 × 10^-10^). Concordantly, the average number of RefSeq genes predicted to be disrupted by genomic deletions was also 1.6-fold higher in cases compared with controls (7.3 vs. 4.7; *P* = 2 × 10^-8^). By comparison, there was no significant difference observed between cases and controls when the number of copy number gains overlapping genes (*P* = 0.1), and the number of genes overlapping copy number gains (*P* = 0.5) were measured.

To determine whether CNV size contributed to genomic burden we calculated the genomic distance between the start and end probes of each PennCNV call. These data showed that there was a slight decrease in the average size of CNVs in cases compared with controls (59.4 kb vs. 65.7 kb; *P* = 0.001); however, there was no statistically significant difference in the average size of the CNVs between the groups when considering copy number type (deletion or duplication) (Table 2). Thus, the enrichment of genes disrupted by genomic deletions in cases of early breast cancer is the result of increased frequency rather than the size of these variants.

**Table 2:** Size of CNVs in breast cancer cases and controls across the whole genome, and overlapping genomic features

CNV type	Mean size of CNVs (kb)		Difference in means (kb)		Case/control ratio	*P* value
	Cases (*n* = 200)	Controls (*n* = 293)	Case - controls	95% CI		
All	59.4	65.7	-6.3	-10.1, -2.5	0.90	0.001
Deletions	42.9	42.7	0.2	-3.0, 3.4	1.00	0.89
Gains	10.1	10.0	0.1	-7.7, 10.3	1.01	0.77

*CNV* copy number variation

### Rare CNVs in cases of early-onset breast cancer and in controls

To examine the prevalence of rare CNVs, RefSeq gene regions containing five or more CNVs (>1% frequency in the study cohort) were excluded from downstream analyses. By this process, all remaining rare CNVs assessed in this study overlapped RefSeq gene regions. The number of rare deletions in the cases were twofold greater than in controls (1.6 vs. 0.8; *P* = 5 × 10^-4^), but there was no statistically significant difference in copy number gains between the two groups (*P* = 0.6) (Table 3). The average number of RefSeq genes predicted to be disrupted by genomic deletions was 1.8-fold higher in cases compared with controls (2.0 vs. 1.2; *P* = 5 × 10^-3^). The ratio of genes disrupted per rare deletion event in each sample was 1.2 in cases and 1.4 in controls (data not shown). There was no significant difference in ratios between the two groups (*P* = 0.1). Together these results suggest that in most samples rare deletions are disrupting a single gene, and such events in cases of early-onset breast cancer were twice as common as in controls.

**Table 3:** Frequency of rare CNVs (<1% frequency) and overlapping genes in breast cancer cases and controls

Genomic feature	Total frequency		Mean frequency		Difference in means		Case/control ratio	*P* value
	Cases (*n* = 200)	Controls (*n* = 293)	Cases	Controls	Case - controls	95% CI		
Rare CNVs (<1%) overlapping genes								
All	513	491	2.6	1.7	0.9	0.3, 1.5	1.5	0.002
Deletions	327	243	1.6	0.8	0.8	0.4, 1.3	2.0	0.0005
Gains	186	248	0.9	0.8	0.1	-0.2, 0.4	1.1	0.6
Genes overlapping rare CNVs (<1%)								
All	778	749	3.9	2.56	1.3	0.0, 2.6	1.5	0.05
Deletions	403	337	2.0	1.2	0.8	0.3, 1.5	1.8	0.005
Gains	375	412	1.9	1.4	0.5	-0.6, 1.6	1.3	0.4

*CNV* copy number variation

### Rare CNVs disrupting known cancer susceptibility genes

**Fig. 1:**
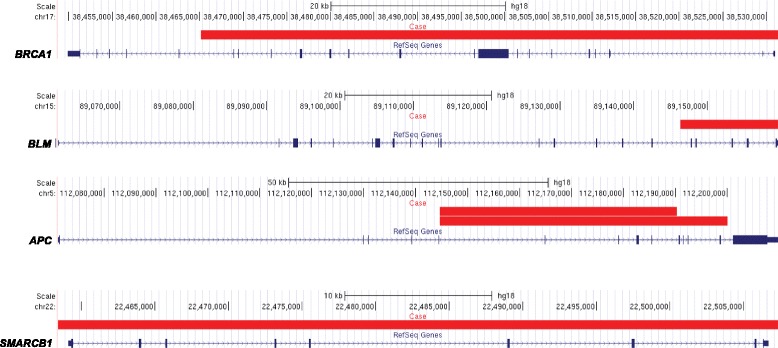
Putative copy number variation (*CNV*) calls overlapping known cancer susceptibility genes in five cases of early-onset breast cancer. UCSC Genome Browser screenshots (NCBI36/hg18) show the location of the CNVs (*solid red rectangles*) in relation to each gene. *RefSeq* reference sequence

Annotation of CNVs in cases and controls against genes known to be involved in cancer-associated syndromes (Additional file 3: Table S3) revealed four cases with deletions in *APC* (*n* = 2), *BRCA1* (*n* = 1) and *BLM* (*n* = 1), and one case with a duplication overlapping *SMARCB1* (*n* = 1). One control was also found to harbour a putative deletion overlapping *APC* (Fig. 1). Subsequent evaluation with qPCR confirmed deletions overlapping *BLM* and *BRCA1,* the duplication overlapping *SMARCB1,* but none of the deletions overlapping the *APC* locus (two cases and one control) (Additional file 3: Table S4). Each validated CNV was shown to overlap one or more of the coding exons within the respective cancer susceptibility gene, suggesting a deleterious effect on the encoded proteins. CNVs potentially associated with risk of breast cancer did not overlap any of the genes that are commonly included in breast cancer predisposition gene panel tests (Additional file 4: Table S5). The *BLM* deletion was genotyped in relatives of the affected proband and identified two additional carriers of deletion but none were affected with breast cancer and an (obligate) non-carrier of this deletion had had breast cancer diagnosed at age 42 years (Additional file 1: Figure S3).

### Genome-wide CNV association study

To identify other new genomic loci contributing to breast cancer, we applied a gene-centric-based approach that defines copy number events by their location within a RefSeq gene region and compares the frequency of these events in cases and controls. Thus, genes overlapping CNVs identified in case and control cohorts were subsequently assigned status as DNA loss or DNA gain. The odds ratio estimates for the association between CNV status and risk of breast cancer are presented in Table 4. Deletions in three genes were associated with increased risk of breast cancer (*DOCK5*, *P* = 3 × 10^-3^; *OR4C11, P* = 2 × 10^-2^; and *OR4P4, P* = 3 × 10^-2^ *).* qPCR did not confirm the small 646-base-pair deletion at the *DOCK5* locus (Table 4). The CNV was predicted by six probes mapping to a 1.4 kb simple tandem repeat region (chr8:25,073,452-25,074,806; GRCh37/hg19). In contrast, deletions were overlapping the *ORC4C11* and *OR4P4* loci were verified by qPCR (Table 4; Additional file 3: Table S4).

**Table 4:** Common copy number changes in RefSeq genes over-represented in early-onset breast cancer cases

Gene	Type	Cases (*n* = 200)	Controls (*n* = 293)	OR	95% CI	*P*	*P*_adjust_^a^	qPCR verified^b^
*DOCK5*	Deletion	29	7	6.9	2.9, 19.0	5 × 10^-7^	3 × 10^-3^	0% (0/4)
*DOCK5*	Gain	3	29	0.1	0.0, 0.5	1 × 10^-4^	NS	0% (0/4)
*OR4C11*	Deletion	71	51	2.6	1.7, 4.1	7 × 10^-6^	2 × 10^-2^	83% (5/6)
*OR4P4*	Deletion	82	66	2.4	1.6, 3.6	2 × 10^-5^	3 × 10^-2^	100% (8/8)
*UGT2B17*	Deletion	57	45	2.2	1.4, 3.5	6 × 10^-4^	NS	Not tested
*OR4C6*	Deletion	62	50	2.2	1.4, 3.4	4 × 10^-4^	NS	Not tested
*OR4S2*	Deletion	65	54	2.1	1.4, 3.3	4 × 10^-4^	NS	Not tested

^a^Multiple testing with a false discovery rate of 5% using the method of Benjamini and Yekutieli. ^b^Percentage of copy number variation (CNV) positive samples tested (number of positive samples/number of samples tested). *RefSeq* reference sequence, *CI* confidence interval, *NS* not significant, *OR* odds ratio, *qPCR* quantitative polymerase chain reaction

## Discussion

In comparison with the large amount of single nucleotide variant data available from breast cancer studies, the contribution of inherited copy number variation to breast cancer risk remains relatively understudied. To our knowledge, this is the largest genome-wide CNV analysis of early-onset (<40 years of age) breast cancer in a population-based study. Our results suggest that CNV frequency (or CNV load) may be associated with breast cancer risk, which is consistent with non-statistically significant data from a previous study of cases of familial and early-onset (<40 years of age) breast cancer [5]. Moreover, our study showed that on average, women with early-onset breast cancer carried one extra deletion within their genome that overlapped a coding gene.

Consistent with our data, a recent analysis of the Exome Aggregation Consortium (ExAC) database showed that the average human genome contains 2.1 rare (<0.5%) CNVs (0.8 deletions, 1.3 duplications) that intersect at least one protein-coding gene [31]. These results are similar to those from controls analysed in this study in which there were 2.6 rare (<1%) CNVs (1.2 deletions, 1.4 duplications) that intersect at least one protein-coding gene. The slightly larger values seen in our study might be explained by the less stringent criterion we used for defining a rare CNV. A possible reason for the larger number of deletions seen in the cases may be related to chemotherapeutic and/or radiological treatment effects on patients. However, in contrast to deletions we observed a reduced total number of duplications in cases compared with controls, and no significant difference between groups when assessing rare CNVs. Such a trend is difficult to explain if the genomic rearrangements are a result of treatment alone. Our results were not directly comparable with results from three other breast-cancer-related studies [5–7] that used a different and more stringent approach to define rare CNVs, that is those showing no overlap or minimal overlap with CNVs listed in the Database of Genomic Variants.

Our study identified a deletion overlapping the known breast cancer susceptibility gene, *BRCA1*, in a woman diagnosed with infiltrating ductal carcinoma of the breast (grade 3) at the age of 39 years, who did not have a family history of the disease and did not have any relatives participating in our study. A rare deletion overlapping the Bloom syndrome RecQ-like helicase gene, *BLM,* was also detected in a patient and some of her family members. Although data were limited for segregation analysis, our results are consistent with the previous finding that *BLM* mutations are associated with a perhaps more moderate increased risk of breast cancer [32]. These data are consistent with this rare allele being associated with a low-to-moderate risk of breast cancer but our study did not have the capacity to formally address or measure a possible link to breast cancer risk. No other women in this study had CNV disruption to cancer susceptibility gene(s). Thus, it remains unclear whether the additional genes disrupted by rare deletions in cases have a causal role in breast cancer risk.

A genome-wide association study of the cases and controls identified deletions overlapping three gene regions (*DOCK5,* 6.9-fold, *P* = 0.003*; OR4C11,* 2.6-fold, *P* = 0.02, and *OR4P4,* 2.4-fold, *P* = 0.03) that were associated with an increased risk of breast cancer after accounting for multiple testing (Additional file 4: Table S5). Assessment of these regions in a subset of samples using orthogonal technology verified the deletions overlapping the olfactory receptor genes, *OR4C11* and *OR4P4,* but not *DOCK5. OR4C11* and *OR4P4* are located at 11q11 and have been previously found to overlap a common bi-allelic deletion [33, 34]. Of note, deletions overlapping *OR4C6* and *OR4S2* that were not associated with breast cancer risk after multiple testing (Table 4) are located adjacent to the *OR4C11/OR4P4* gene locus. To our knowledge, variants overlapping the *OR4C11*/*OR4P4* gene locus have not previously been shown to be associated with breast cancer risk. It is unclear how this locus would have a causative role in breast cancer development, although 11q deletions are commonly found in breast tumours, particularly those classified as having a high histological grade [35]. A review of two breast tumour datasets from Pereira et al. (*n* = 1980) and The Cancer Genome Atlas (*n* = 960) showed that expression of *OR4C11* and *OR4P4* did not correlate with copy number loss (data not shown) [36, 37], although this locus may harbour regulatory element(s) that control key genes from long range.

CNVs overlapping more than 100 genes have been found exclusively or at a greater frequency in cases of familial and/or early-onset breast cancer; however, none of these loci have been identified in more than one study [8]. Large-scale studies of women with early-onset breast cancer are now required to better understand the contribution of germline CNVs to breast cancer risk. Such CNV-based studies are now possible by utilising available SNP genotyping data generated by massive genome-wide association studies that include cases of early-onset breast cancer [38, 39].

## Conclusions

We report that the frequency of rare CNVs may be associated with breast cancer risk, and that compared with controls, patients with early-onset breast cancer carried one extra deletion within their genome that overlapped a coding gene. A genome-wide analysis of CNVs identified deletions at the *OR4C11/OR4P4* locus that were also associated with breast cancer risk. Larger studies are required to further investigate these possible associations to understand the role of CNVs in the development of breast cancer.

## Additional files

Additional file 1: Figure S1. Study design for CNV discovery, quality control and analysis. **Figure S2.** The protocol use to define RefSeq gene boundaries. **Figure S3.** *BLM* deletions identified in a familial breast cancer pedigree. Male and female individuals are represented by squares and circles, respectively. The index case patient, who underwent genome-wide CNV profiling, is indicated by an *arrow*. Individuals with breast cancer are represented by *closed circles*. Other cancers are indicated by black shading in the lower left quadrant. Age at death, last known diagnosis or cancer diagnosis is indicated where known. Copy number genotype at the *BLM* locus is noted as *BLM* deletion (*BLM del*) or wildtype (*wt*) copy number. (DOCX 30 kb)
Additional file 2: Table S1. CNV data for cases and controls. Chromosome coordinates given according to Hg18. (XLSX 819 kb)
Additional file 3: Table S2. Taqman assays used for CNV assessment at 7 gene loci. **Table S3.** Cancer-predisposing genes disrupted by rare CNVs. **Table S4.** Results of Taqman assays carried across 7 gene loci. (DOCX 18 kb)
Additional file 4: Table S5. Results from a genome-wide association analysis of CNVs overlapping gene loci. (XLSX 205 kb)

## Abbreviations

BAF: B allele frequency
CI: Confidence interval
CNV: Copy number variation
Kb: kilobase
LRR: Log R ratio
MAF: Minor allele frequency
OR: Odds ratio
qPCR: Quantitative polymerase chain reaction
RefSeq: reference sequence
SNP: single nucleotide polymorphism
