# Supplementary material for: Increased genomic burden of germline copy number variants is associated with early onset breast cancer: Australian breast cancer family registry
Source: Breast Cancer Res. 2017 Mar 16;19:30. doi: 10.1186/s13058-017-0825-6 (PMC5356248; doi:10.1186/s13058-017-0825-6)
Supplement: Additional file 1: Figure S1. — Study design for CNV discovery, quality control and analysis. Figure S2. The protocol use to define RefSeq gene boundaries. Figure S3. BLM deletions identified in a familial breast cancer pedigree. Male and female individuals are represented by squares and circles, respectively. The index case patient, who underwent genome-wide CNV profiling, is indicated by an arrow. Individuals with breast cancer are represented by closed circles. Other cancers are indicated by black shading in the lower left quadrant. Age at death, last known diagnosis or cancer diagnosis is indicated where known. Copy number genotype at the BLM locus is noted as BLM deletion (BLM del) or wildtype (wt) copy number. (DOCX 30 kb) [file 13058_2017_825_MOESM1_ESM.docx]

**Figure S1.** Study design for CNV discovery, quality control and analysis

**Figure S2**. The protocol use to define RefSeq gene boundaries

**Figure S3**. *BLM* deletions identified in a familial breast cancer pedigree. Males and females are represented by squares and circles, respectively. The index case who underwent genome-wide CNV profiling is indicated by an arrow. Breast cancer-affected individuals are represented with filled circles. Other cancers are indicated by bottom left quadrant filled in. Age at death, last known diagnosis or cancer diagnosis is indicated where known. Copy number genotype at the *BLM* locus is noted as *BLM* del (deletion) or wildtype (wt) copy number.
