# Supplementary material for: Increased genomic burden of germline copy number variants is associated with early onset breast cancer: Australian breast cancer family registry
Source: Breast Cancer Res. 2017 Mar 16;19:30. doi: 10.1186/s13058-017-0825-6 (PMC5356248; doi:10.1186/s13058-017-0825-6)
Supplement: Additional file 3: Table S2. — Taqman assays used for CNV assessment at 7 gene loci. Table S3. Cancer-predisposing genes disrupted by rare CNVs. Table S4. Results of Taqman assays carried across 7 gene loci. (DOCX 18 kb) [file 13058_2017_825_MOESM3_ESM.docx]

**Table S2**. Taqman assays used for CNV assessment at 7 gene loci

| **Assay ID** | **Target Loci** | **Assay Location** | **CNV region (Hg19 build)** |
| --- | --- | --- | --- |
| Hs03563250_cn | *APC* | chr5:112139984 | chr5:112116730-112162413 |
| Hs05380780_cn | *BLM* | chr15:91357302 | chr15:91345527-91358862 |
| Hs05488646_cn | *BRCA1* | chr17:41225475 | chr17:41223727-41224748 |
| Hs06243508_cn | *DOCK5_upstream* | chr8:25072454 | chr8:25072334-25075246 |
| Hs05034780_cn | *DOCK5_downstream* | chr8:25075096 | chr8:25072334-25075246 |
| Hs01985934_cn | *OR4C11* | chr11:55371563 | chr11:55371024-55371849 |
| Hs00972795_cn | *OR4P4* | chr11:55406375 | chr11:55405834-55406772 |
| Hs05575788_cn | *SMARCB1* | chr22:24158649 | chr22:24129150-24176705 |

| **Table S3.** Cancer-predisposing genes disrupted by rare CNVs | | | | |  |  |
| --- | --- | --- | --- | --- | --- | --- |
| **Gene** | **Locus** | **Cancer syndrome** | **Cancer type** |  |  |  |
| *APC* | 5q21–q22 | Adenomatous polyposis coli; Turcot syndrome | Colorectal, pancreatic, desmoid, hepatoblastoma, glioma, other CNS |  |  |  |
| *BMPR1A* | 10q22.3 | Juvenile polyposis | Gastrointestinal polyps |  |  |  |
| *BRCA1* | 17q21 | Hereditary breast/ovarian cancer | Breast, ovarian |  |  |  |
| *BRCA2* | 13q12.3 | Hereditary breast/ovarian cancer | Breast, ovarian, pancreatic, leukemia (FANCB, FANCD1) |  |  |  |
| *CDH1* | 16q22.1 | Familial gastric carcinoma | Gastric, breast |  |  |  |
| *CDKN1B* | 12p13.1 | Multiple endocrine neoplasia type IV | Pituitary tumor, testicular tumor |  |  |  |
| *CDKN2A* | 9p21 | Familial malignant melanoma | Melanoma, pancreatic |  |  |  |
| *CHEK2* | 22q12.1 | Familial breast cancer | Breast, prostate |  |  |  |
| *CREBBP* | 16p13.3 | Rubinstein–Taybi syndrome | Nervous system, brain, leukemia |  |  |  |
| *CYLD* | 16q12.1 | Brooke–Spiegler syndrome, familial cylindromatosis, multiple familial trichoepithelioma | Multiple skin appendage tumors |  |  |  |
| *EPCAM* | 2p21 | Lynch syndrome | Colorectal, endometrial |  |  |  |
| *EXT1* | 8q24.11–q24.13 | Multiple exostoses type 1 | Exostoses, osteosarcoma |  |  |  |
| *EXT2* | 11p12–p11 | Multiple exostoses type 2 | Exostoses, osteosarcoma |  |  |  |
| *FANCA* | 16q24.3 | Fanconi anemia A | Acute myeloid leukemia |  |  |  |
| *FH* | 1q42.1 | Hereditary leiomyomatosis and renal cell cancer | Lieomyomatosis, renal |  |  |  |
| *FLCN* | 17p11.2 | Birt–Hogg–Dubé syndrome | Renal cell carcinoma |  |  |  |
| *GPC3* | Xq26 | Simpson–Golabi–Behmel syndrome | Wilms’ tumors |  |  |  |
| *CDC73* | 1q31.2 | Hyperparathyroidism–jaw tumor syndrome | Parathyroid carcinoma, renal cell carcinoma |  |  |  |
| *JAG1* | 20p12 | Alagille syndrome | Hepatocellular carcinoma, papillary thyroid carcinoma |  |  |  |
| *MEN1* | 11q13 | Multiple endocrine neoplasia type 1 | Parathyroid adenoma, pituitary adenoma, pancreatic islet cell, carcinoid |  |  |  |
| *MLH1* | 3p21.3 | Lynch syndrome, Turcot syndrome | Colorectal, endometrial, ovarian, CNS |  |  |  |
| *MSH2* | 2p22–p21 | Lynch syndrome | Colorectal, endometrial, ovarian |  |  |  |
| *MSH6* | 2p16 | Lynch syndrome | Colorectal, endometrial, ovarian |  |  |  |
| *NF1* | 17q11.2 | Neurofibromatosis type 1 | Neurofibroma, glioma |  |  |  |
| *NF2* | 22q12.2 | Neurofibromatosis type 2 | Meningioma, acoustic neuroma |  |  |  |
| *NSD1* | 5q35.3 | Sotos syndrome | Increased risk of benign or malignant tumors, including neuroblastoma and gastric carcinoma |  |  |  |
| *PMS2* | 7p22 | Lynch syndrome, Turcot syndrome | Colorectal, endometrial, ovarian, medulloblastoma, glioma |  |  |  |
| *PRKAR1A* | 17q23–q24 | Carney complex | Myxoma, endocrine, papillary thyroid |  |  |  |
| *PTCH1* | 9q22.3 | Nevoid basal cell carcinoma syndrome | Skin basal cell, medulloblastoma |  |  |  |
| *PTEN* | 10q23.31 | Cowden disease; Lhermitte–Duclos syndrome | Breast cancer, leukemia, renal cell adenocarcinoma, neuroendocrine carcinoma, Merkel cell carcinoma |  |  |  |
| *RB1* | 13q14.1–q14.2 | Familial retinoblastoma | Retinoblastoma, sarcoma, breast, small cell lung |  |  |  |
| *RUNX1* | 21q22.12 | Familial platelet disorder | Acute myeloid leukemia |  |  |  |
| *SDHB* | 1p36.1–p35 | Familial paraganglioma | paraganglioma, pheochromocytoma |  |  |  |
| *SDHC* | 1q21 | Familial paraganglioma | Paraganglioma, pheochromocytoma |  |  |  |
| *SDHD* | 11q23 | Familial paraganglioma | Paraganglioma, pheochromocytoma |  |  |  |
| *SMAD4* | 18q21.2 | Juvenile polyposis syndrome | Colon, stomach, small bowel and pancreas |  |  |  |
| *SMARCB1* | 22q11 | Rhabdoid predisposition syndrome | Malignant rhabdoid |  |  |  |
| *STK11* | 19p13.3 | Peutz–Jeghers syndrome | Jejunal harmartoma, ovarian, testicular, pancreatic |  |  |  |
| *TP53* | 17p13.1 | Li-Fraumeni syndrome | Breast, sarcoma, adrenocortical carcinoma, glioma, multiple other tumor types |  |  |  |
| *TSC1* | 9q34 | Tuberous sclerosis 1 | Hamartoma, renal cell |  |  |  |
| *TSC2* | 16p13.3 | Tuberous sclerosis 2 | Hamartoma, renal cell |  |  |  |
| *VHL* | 3p26–p25 | von Hippel–Lindau syndrome | Renal, hemangioma, pheochromocytoma |  |  |  |
| *WT1* | 11p13 | Denys–Drash syndrome, Frasier syndrome, Familial Wilms tumor | Wilms tumor |  |  |  |

**Table S4**. Results of Taqman assays carried across 7 gene loci.

| **Assay** | **Accuracy** | **Duplication** | **Deletions** |
| --- | --- | --- | --- |
| *APC* | 0% (0/3) | - (0/0) | 0% (0/3) |
| *BLM* | *100% (1/1)* | - (0/0) | *100% (1/1)* |
| *BRCA1* | *100% (1/1)* | - (0/0) | *100% (1/1)* |
| *DOCK5_ upstream* | 0% (0/4) | - (0/0) | 0% (0/4) |
| *DOCK5_ downstream* | 0% (0/4) | - (0/0) | 0% (0/4) |
| *OR4C11* | *83% (5/6)* | - (0/0) | *83% (5/6)* |
| *OR4P4* | *100% (8/8)* | - (0/0) | *100% (8/8)* |
| *SMARCB1* | *100% (1/1)* | *100% (1/1)* | - |
